# Using EMDR to treat intimate partner relationship break-up issues

**DOI:** 10.3389/fpsyg.2022.971856

**Published:** 2022-09-29

**Authors:** Ainhoa Rodríguez-Garay, Dolores Mosquera

**Affiliations:** ^1^Barnapsico, Barcelona, Spain; ^2^Instituto de Investigación y Tratamiento del Trauma y los Trastornos de la Personalidad (INTRA-TP), A Coruna, Spain

**Keywords:** eye movement desensitization and reprocessing, relationship, traumatic experience, attachment, break-ups, abuse

## Abstract

The Break-Up Aid Procedure (BUAP) is a proposed EMDR intervention designed to address the difficulties that some patients experience when trying to overcome a complex break-up. The procedure aims to not only target the consequences of the break-up, but also the difficulties that may be present in starting a new relationship. The latter is often the case when there are either unresolved consequences from the previous break-up or untreated early adverse experiences. By structuring the treatment in three sequential stages (outlined in this article) and including interventions and adaptations to the standard EMDR protocol, we aim to facilitate the clinician’s therapeutic approach to intervening for this specific presenting concern and maximizing success.

## Introduction

A break-up from an intimate partner relationship is one of the most difficult events for individuals to manage. According to a scale measuring vital stressful events (*The Holmes-Rahe Stress Inventory*; Holmes and Rahe, 1967), divorce and separation in marriage rank as one of the top three stressful events in an individual’s life. This stressor is only followed by the death of the married partner. A breakup, in and of itself is demanding for individuals but it can become increasingly complicated if either the end of the relationship is traumatic, or the person has previously lived through adverse affective experiences; particularly if those took place during early stages of life.

In both instances, the information processing system that allows us to reach “adaptive solutions” when we suffer difficult experiences could get stuck and affect the natural grief process (Shapiro and Forrest, 2009). This could justify a psychological intervention using Eye Movement Desensitization and Reprocessing (EMDR; Shapiro, 1995).

The intervention introduced in this article, called Break-Up Aid Procedure (BUAP), adapts the standard EMDR Protocol to the treatment of individuals experiencing a relationship break-up process that is complex. Specifically, it is designed to address the difficulties encountered during the grief process or the emotions that are activated after the end of the intimate relationship. With this procedure, our hope is to address a two-fold purpose: first, help patients engage in their grief process in an adaptive manner; and second, allow the crisis itself to serve as a point of departure to improve the patient’s capacity to establish and maintain positive and functional relationships in the future. Moreover, in this article, additional interventions are described to support individuals who will struggle to break away from a harmful relationship or one which they no longer wish to maintain.

## Stages of intervention in BUAP

A break-up from an intimate partner relationship tends to generate, amongst others: the loss of a person’s secure base; subsequent anxiety about the separation; and elevated emotional and physiological activation that may, initially, be very difficult to manage. With the goal of focusing on the main needs of the patient during each stage of recovery from the break-up, and structure the psychological intervention, we propose a three-stage approach.

The proposed intervention modifies the order in which the three-pronged standard EMDR protocol (Shapiro, 1995, 2001) is delivered. We begin working on the present and most recent past, instead of the past as we would traditionally do in the standard protocol. We therefore propose that therapists adopt the following approach (Table 1).

**Table 1:** Break-Up Aid Procedure^1^

**Stages of the intervention**	**Therapeutic goals**	**Resources and specific interventions**
**Stage 1**	In case of maltreatment and/or abuse, gathering of a comprehensive understanding of the victim´s difficulties from a domestic violence perspective, including safety issues and frequent distortions during initial contact.	Informing the patient, guaranteeing safety (self-protection and external protection), addressing guilt, identifying factors that make it difficult for the client to end the relationship in order to intervene on stuck points.
	EMDR Stage 1. Progressive history gathering; extending to stage 2 of the treatment.	In this stage, specific information about the couple's relationship and the break-up is gathered.
	Stabilization. Providing the patient with needed resources and skills that can help with the present difficulties he or she is facing due to the consequences of the break-up.	Psychoeducation and skills acquisition: Introducing adaptive information on couple break-up, manipulative behaviors of abusers, emotional regulation resources, self-care assessment and intervention, etc.
		Installation of EMDR resources, such as 4 elements, resources to increase motivation to end the relationship, future moment having overcome the break-up, etc. (Popky, 1994, 2005; Hase, 2010)
	Reducing physiological and emotional activation.	R-TEP (intervention on the present and the recent past).
	Addressing specific difficulties in this population, such as idealization, blocking beliefs and emotional dependency experienced as an addiction.	Work on idealized image/s or memories (Mosquera, Knipe, 2016, 2017; Mosquera, 2022).
		Intervention on blocking beliefs (Knipe, 2019).
		Desensitizing the urge related to the addictive behavior (Popky, 2005; Hase 2006).
**Stage 2** Addressing persistent symptoms after the break-up and preparation of the person to establish healthy relationships.	Continuing with progressive history gathering for a more specific understanding of the dynamics that are getting in the way of improvement. Collecting information about persistent symptoms and their relationship with present difficulties.	Establishing a link between persistent symptoms, present difficulties, and past unresolved adverse and traumatic experiences. Inquiring further into the past, especially attachment, early experiences and previous relationships.
	Assessing consequences and difficulties to identify and work with specific skills that the person needs to acquire. __________________________________________ Identification and reprocessing targets with EMDR	Building adaptive information. Psychoeducation (e.g. unrealistic expectations about relationships, need for control...)
		Specific resources (e.g., acquisition of social skills, anxiety management, identification of dysfunctional behaviors...) __________________________________________________ Completing target selection from: - Factors of vulnerability to revictimization. - Triggers and persistent symptoms - Identified relationship problems related memories
**Stage 3** Accompaniment during the initiation and/or maintenance of new relationships.	Identification difficulties in initiating and/or maintaining healthy relationships	Focusing on communication style, empathy, emotional regulation, anxiety management, etc.
	Identification and reprocessing triggers that arise as the person initiates a new relationship.	Reprocessing triggers that interfere with the capacity to have a healthy relationship focused on the present due to the link with unresolved adverse and traumatic memories. Future templates when needed.

^1^When we use the term Phase we refer to the Phases of EMDR Therapy and Standard Protocol. When we use the term Stage we refer to the Break-up Aid Procedure (BUAP)

### BUAP stage 1: Working with the present situation and recent past

During the initial sessions, assess which of the following interventions may be needed:

While gathering the patient’s history, focus on the issues which led to the break-up and the consequences these had and/or are having on the individual’s present functioning.
If the therapist determines that the individual is lacking the necessary resources to cope, then one would carry out resource installation and provide adequate psychoeducation.
If required the therapist would use EMDR’s recent traumatic episode protocol (R-TEP; Shapiro and Laub, 2008) to reduce the patient’s emotional and physiological activation.
Other EMDR procedures may be used to address concrete aspects of the relationship break-up, or the need to stay away from an ex-partner. This may include working with an idealized image (Mosquera and Knipe, 2016, 2017); or the subjective sensation of addiction to the ex-partner which may be addressed using the Craving Extinguished protocol for the addicted memory (Hase, 2010).
When the grief process becomes complex, the therapist identify what impedes the grief from progressing through its natural course to determine how to intervene.

We suggest that for patients where abuse was present in the relationship, the therapist also conducts the following interventions:

Prioritize the patient’s safety by conducting a thorough risk assessment. Inform the patient about resources that exist in their local community and from which they may benefit. This will allow for the various psychosocial, legal, and protective measures to be put in place. Do this independently from whether the patient continues therapy or not. Moreover, assess if the patient is putting adequate self-protective measures in place. It is particularly important that the therapy consult be perceived as a safe space.
Turn your attention to whether the patient is struggling to end the relationship. The main goal would be to identify which factors (e.g., fear, ambivalence, financial or psychological dependence, subjective sensation of addiction to the partner or to another component of the relationship, etc.) are blocking the patient’s ability to make adaptive decisions for themselves, so we can decide how to intervene.
Determine whether the patient is assigning guilt appropriately. To carry out this intervention ensure that the victim can be freed from guilt, and that the responsibility may be adequately assigned to the person who is perpetrating the abuse and/or violence.
Accompany the victim in the painful and complex process of gaining full awareness of the damaging aspects of their relationship and of the negative aspects of their partner or ex-partner. It is important that the victim understands that their current sense of malaise is mainly a result of the traumatic bond they maintain, or maintained, with their aggressor.
Support the victims by helping them discover that they have other options and that it is possible to overcome the traumatic experiences they lived through. This is done while developing new expectations for the future that are not conditioned by their past.

### BUAP stage 2: Process specific past experiences

In this second stage, of our proposed interventions, therapists would process the memories that mediate the symptomatology and those related to the difficulties to overcome the break-up and/or establish healthy and functional relationships. To do so, we propose the following interventions:

Capture the relationship memories that have been dysfunctionally stored; including those from childhood as well as those from previous intimate partner relationships.
Determine which abilities the patient needs to develop and the specific needs they have for psychoeducation.
Process identified targets as well as their current triggers.

In cases where there has been abuse, the therapist needs to address those aspects that increase the patient’s vulnerability to being re-victimized (e.g., prior experiences of victimization that have not been processed; difficulties in maintaining personal boundaries; excess of naivete in relationships; etc.).

### BUAP stage 3: Identify difficulties in starting or maintaining a future intimate partner relationship

Interventions during this stage of the suggested procedure are designed to address those difficulties that will emerge as the patient begins to make real changes in their interpersonal relationships and contemplates the beginning of a new intimate partner relationship.

With each difficulty that is noted, work through the standard three-pronged protocol. Begin with current triggers and then work on memories from the past that continue to activate symptoms. You may also need to work on other relevant memories that may not have been previously identified. Close the work with future templates, which will help you discover aspects that have not been resolved so they can be processed.
Help the patient develop the necessary abilities to maintain intimate partner relationships that are healthy, satisfying, and balanced. This aspect is especially important for those individuals who have not been able to establish good relationships throughout their life.

While this work structure has been described as though the therapeutic process is linear, in practice, it may not necessarily work out this way. On occasion, the patient may either be unable or unwilling to work through all the stages. For others, it may be unnecessary to work through any of these stages. For others yet, the stages may overlap (e.g., when the individual starts a relationship with an intimate partner before grieving or working through the difficulties that underlie the reason why their relationships are often affected).

## Phases of intervention using EMDR

When a patient seeks consultation for difficulties they experience when confronting the break-up from an intimate partner relationship, the therapist will guide them through the treatment structure of the eight phases of EMDR (Shapiro, 2001). However, some adaptations are strongly suggested when working on this specific presenting concern (Table 2).

**Table 2:** Comparing EMDR’S standard protocol and BUAP.

Standard protocol	Break ups aid procedure
8 phases	The 8 phases of the standard protocol are maintained, but the 1st phase (conceptualization of the case) is carried out progressively, exploring early experiences as the patient is more stabilized.
Order of interventions: - Past (traumatic memories) - Present (triggers) - Future (future templates)	A 3-stage intervention is proposed: - First stage: Beginning with present and recent past until the person is more stabilized. - Second and third stage: persistent difficulties are addressed using the standard protocol.
Reprocessing of traumatic memories.	Reprocessing of pathogenic memories, including early traumatic memories and desensitization of defenses and dysfunctional positive affect.

Having a structure for intervention is not only useful in the exploration and understanding of the patient’s presenting concern, but also in treatment planning and application. Despite this, we must maintain a framework that is flexible enough to not lose sight of the individual, their experiences, doubts, needs, fears, strengths, and vulnerabilities. This flexibility will allow the development of the therapeutic process in a way that supports any interferences or difficulties that may emerge along the way. One of the objectives that will guide the interventions will be our ability to understand the patient’s concrete difficulties and limitations, what kept them from overcoming these issues, and how these are connected to their life history.

## EMDR phase I: Patient history

When the primary demand being faced by the patient revolves around a break-up from an intimate relationship partner, Phase 1 of EMDR (i.e., collection of the patient’s history) will need to be adapted. The patient’s current preoccupation with the break-up will interfere with their ability to focus on sharing the underlying problems that would be convenient to address. Therefore, the history taking should be adapted to the needs and resources that the patient has in that moment. This may mean that the case conceptualization will need to be developed in a progressive manner throughout the therapeutic process, making it gradual and continuous.

It is recommended that one begins by focusing more on the present and in understanding the characteristics of the patient’s current or past relationship, as well as the factors that may interfere in their ability to overcome the break-up. As the emotional and physiological activation starts to subside and the main interferences in their day-to-day level of functioning begin to decrease, we will be able to explore more aspects of the patient’s history. It is then that one can begin to identify the adverse experiences (often during early developmental stages) that gave way to the current difficulties, or that serve to maintain them. During this phase, you will also identify the abilities that the patient will need to develop to be able to enjoy healthy relationships.

### Part I: Collection of relationship history and current situation

When treating patients with this presenting concern, you are encouraged to explore the following: (a) conscious and unconscious motives influencing the patient’s choice of intimate partner or ex-partner; (b) the function(s) of the relationship in the patient’s life; (c) factors that brought patient and partner together and separated them; and (d) the impact that the break-up had on the patient’s sense of self and their life. In those cases where the patient is struggling to end the relationship, try to inquire about what is hindering them from letting go; what they will lose with the relationship break-up; what worries them most about ending it; and what aspects of the break-up do they fear most.

Below, we propose various sections that we consider important when collecting information for the case conceptualization:

#### Ending the relationship

Aside from exploring aspects such as who ended the relationship and the motives behind it (e.g., infidelity, lies, betrayal, abuse, violence, etc.), it is important to know if there were previous failed attempts to end the relationship.

##### Motives for leaving the relationship

We encourage the patient to put together a list of the motives that led them to end the relationship; particularly if there had been instances of abuse. This will be useful when there is: reduced motivation to end the relationship; a desire to get in touch with the ex-partner; or an increased presence of an idealized image rather than a real one. The patient may be more willing to access the motives for why they do not want to continue in the relationship if they are aware of these issues. This information offers some insight into the relationship dynamics as well as some of the personal characteristics of each of the individuals in the relationship.

##### Ambivalence and internal conflict

To address these aspects we explore the motives for keeping the relationship despite their intention to end it and explore the existence of possible internal conflicts that may interfere in the process of overcoming the break-up. For example, wanting to separate from the intimate partner but not feeling capable of doing so; or oscillating between being clear and feeling sad, angry and/or guilty.

If there the presence of idealization of the partner or of the relationship is encountered, we suggest the exploration of the good moments and the positive memories while evaluating how realistic these might be. The lesser amount of realistic positive memories, the more serious the idealization will be.

Patients who have been victims of abuse may present great ambivalence about the decision to put a definite end to the relationship. This could happen because of feeling incapable, fearful, confused, or guilty. It may also be due to an intense dependency on the partner that they may be idealizing. Generally, victims experience weakness at all levels of functioning because their self-esteem, general mood, and feelings of low self-worth are often impacted. This will be particularly true when they have failed in previous attempts to leave the relationship.

##### Degree of motivation to leave the relationship

The patient is asked to rate their motivation to leave the relationship on a scale from 0 to 100. To process the proposed targets (when there is a subjective sensation of addiction), the patient would ideally offer a rating of 70% or higher in this motivation scale; however, a rating above 50% is sufficient to attempt the intervention (Knipe, 2019).

It is possible to augment the level of motivation through the installation of resources. For example, the therapist might ask: “What would help increase your motivation to end the relationship?” The answer to this question will help identify the resources the patient may need to increase their motivation through installation.

When there is insufficient motivation or the level of motivation does not increase, we can ask the patient what is impeding them from doing so. This will help you determine how to intervene. A way of doing this may be by asking: “What’s good about not having sufficient motivation to end the relationship?” Using the answer to this question we can determine how to address the block that is keeping the patient from ending the relationship or to identify what other resource may be needed.

#### Characteristics of the relationship

Explore the stage of the relationship they currently find themselves in (e.g., falling in love, engaged, mature love, etc.); how the patient and their partner faced changes in each stage of the relationship; and how they functioned around their sexuality. One also briefly explores each of the following aspects of the relationship.

##### Negative events

The patient is encouraged to make a list of the negative events experienced in the relationship with their intimate partner or ex-partner. This will offer valuable information about the characteristics of the relationship. Moreover, this knowledge will be useful in identifying targets or in having material more accessible when the patient is ambivalent.

##### Patterns in the relationship

Attempts are made to identify patterns of conflict that damaged the relationship and affected the patient (i.e., related to their attachment adaptation). This is done to intervene in the present (if necessary and wanted) as well as when patients need support to face future relationships.

##### Interpersonal boundaries

Difficulties in setting and maintaining healthy boundaries with the intimate partner are explored. Difficulties setting boundaries will be particularly problematic if the patient who wants to leave the relationship has a partner who does not want the relationship to come to an end. On the other hand, if the patient is the person who is violating others’ boundaries, theywill need to be challenged empathically to bring this to their awareness.

##### Subjective sensation of addiction to the intimate partner or the relationship

It is possible for the patient to feel that an aspect of the relationship (i.e., affectionate, relational, and/or sexual) represents a strong addictive component, or that the relationship is a dysfunctional bond they do not know how to manage. In assessing this factor, the patient’s sense of urgency to go back to the relationship or to the intimate partner, needs to be evaluated, despite them having made the decision to not do so or having awareness that the relationship is damaging to them.

#### Personal characteristics

The practitioner should briefly inquire about the following aspects related to the personal characteristics of the patient.

##### Tolerance for solitude

If the patient encounters difficulties being alone, they will have difficulties breaking the relationship even when it is dysfunctional. It is also likely that the patient will rely on compensatory behaviors or will desperately seek another partner before grieving the end of the last relationship.

##### Emotional dependence

While it is not recommended that one initially dives into the causes for this type of dependency, it is important that one points it out to the patient, offering them resources to manage it. Doing so will help reduce possible difficulties associated to emotional dependence. Generally, this means that, at a minimum, we will address the concept of differentiation as well as ways to manage and cope with anxiety.

##### Self-care and problematic behaviors

The *Self-Care Scale* (González-Vazquez et al., 2018) may be used to assess self-care practices as part of this assessment, one must explore if the patient is engaging in behaviors to overcome the relationship break-up. Moreover, therapists need to learn whether the patient can manage difficult emotions; whether such emotions create problems for the individual; or whether the patient are inefficacious regulating them. If so, one finds out how this benefit the patient in the short-and long-term so alternative and more beneficial skills and behaviors can be identified.

##### Blocking beliefs

May be identified using the blocking beliefs questionnaire (Knipe, 1998b). While this assessment measure was designed for a therapist and their patient to explore which beliefs are keeping the patient from overcoming an addiction, it can also be useful with other presenting concerns. Once the dysfunctional beliefs have been identified, it will be important to process them and even use bilateral stimulation (BLS) to encourage change of these blocking beliefs (Knipe, 2019).

##### Self-esteem

To evaluate the patient’s current level of self-esteem, it is recommended that the therapist explores the patient’s realistic view of self (Mosquera and Knipe, 2016). On one hand, one can explore if the individual values oneself; if they feel they deserve to be loved; and if they believe their needs should be met. On the other hand, one can explore if the patient has unrealistic demands and expectations in their interpersonal relationships. In patients who have been victims of abuse, one frequently finds that their self-esteem has been negatively affected as a direct consequence of having a traumatic bond; independently of whether they had low self-esteem prior to the relationship or not.

##### Guilt

It is expected that in any grief process feelings of guilt will emerge because of the circumstances of the loss. It is also common for the individual to oscillate between blaming others and blaming oneself. Even though part of the recovery process consists in a person assuming one’s own responsibility and working through it (Shapiro and Forrest, 2009), on occasion, there is a feeling of maladaptive guilt. This internalization of guilt may have already been present during childhood if the patient had inadequate or deficient parents as caregivers (Knipe, 2019) and is now exacerbated by the relationship break-up.

For some abuse victims, feeling (misplaced) guilt could be the last resource available to them to maintain a sense of control over the terrible experiences they lived through. They prefer to feel guilty than to feel impotent or even victimized. On the other hand, those who abuse their partners often try to convince (gaslight) them that they are responsible for what happened. In this manner, they can elude any responsibility over their actions and keep the victim confused and submissive to their desires.

##### Defenses

In this article, we refer to defenses as the psychological mechanisms through which individuals protect themselves – generally in an unconscious way – from painful emotions or from information that contradicts or threatens their core beliefs; especially those about their selfconcept. Defenses are ways of coping that have been useful during given moments, but that have currently stopped working, and keep the individual from adaptive functioning.

During case conceptualization, one needs to explore which psychological defenses are used by the patient to cope with their difficult internal experiences. To this end one examines how they interfere with day-to-day moments as well as during the therapy process. Some of the most common defenses that tend to be present are denial, minimization, avoidance, rationalization, idealization, and perfectionism.

#### Resources to cope with the break-up

Once the patient’s vulnerabilities have been identified, the therapist can begin to explore whether the patient has the necessary positive resources at their disposal to manage their complicated experiences and handle the therapy process. These may include personal strengths such as emotional regulation that is inclusive of positive experiences, social supports, abilities or interests, and healthy activities.

##### Personal strengths

These can be brought to the fore through exploration of prior situations where the patient was able to overcome difficult moments (i.e., other relationship break-ups), or where they felt in control and had the capacity to manage adversities. If there are no networks of adaptive memories, they will need to be constructed therapeutically during the preparation phase (Shapiro, 2012).

##### Emotion regulation

Assess whether the patient has the capacity for emotion regulation. Difficulties in this area are one of the primary reasons why patients can experience grief as intolerable. These patients will resort to behaviors to manage their pain that can be counterproductive. When there are critical difficulties in self-regulation, it will be important to identify the reasons why the patient does not tolerate one’s emotions, or why they fear or experience them as something intolerable. Such patients may be offered psychoeducation to help them gain and develop the tools and abilities that will allow them to connect, identify, validate, and regulate their emotions. On occasion, it will be necessary to identify the memories associated with difficulties in emotion regulation so they can be processed.

##### Social supports

Inquire whether the patient has social supports and whether these individuals validate the patient or not. This is particularly important if the patient has been a victim of violence and/or abuse. If there is invalidation or guilting of the victim, it will be necessary to challenge some of the messages they have received from others and teach them to neutralize them. It may also be necessary to teach them how to set boundaries with those individuals.

Where there has been violence and/or abuse, one of the main tasks will consist in identifying patient characteristics that could make them more vulnerable to damaging relationships or make them a victim of manipulation and/or any type of abuse. For example, it will be necessary to explore in which moment the patient started to become aware of their partner’s negative aspects; why they were not able to notice these before; and whether they had already identified some indicators that interfered in their ability to protect themselves when they were warned that it could be a damaging relationship.

### Part II: Collection of complete history

When the patient is sufficiently stabilized and prepared to face pertinent aspects of their past, we recommend the collection of necessary information to intervene with the three-pronged (past, present, and future) standard EMDR protocol. However, the order in which to process the targets will depend on each concrete case.

When collecting history, one explores early experiences as well as relationship experiences throughout one’s lifespan. Present triggers need to be identified; particularly those that interfere in interpersonal relationships and those that may keep the patient from establishing future, healthy relationships.

#### Early relationships

##### Attachment style

In Siegel (2012) words: “It is probable that the first attachment experiences in life directly shape cortical processes implicated in corporal regulation; one’s capacity to communicate with others; emotional equilibrium; flexibility; empathy; self-understanding; and one’s capacity to self-soothe fearful states (p. XVII).” For this reason, as part of assessing the patient’s adult attachment style, the therapist needs to identify the type of attachment adaptation the patient had with each of the primary caregivers during infancy. In patients where the attachment to the mother was insecure, one would explore if there was an incident during the perinatal stage that led to physical and/or emotional separation that could have affected the mother–child bond. In turn, this could have affected the development of attachment, for which the mother–child bond is considered a necessary, but not sufficient condition (Madrid, 2012).

##### Family genogram

Doing a family genogram has a double function: evaluation and intervention. It facilitates the patient gaining awareness of the importance of some trans-generational difficulties; behavioral patterns; and dynamics in interpersonal relationships that may affect their relationship with an intimate partner. Understanding how this transmission of trans-generational difficulties occurs, may bring insight to the patient about their parents’ problematic behaviors and/or those of other family members and/or caregivers. This can help some patients gain perspective and may even bring healing to some. It may also be useful to explore if there were hierarchical problems in the family of origin. For example, setting boundaries; accepting authority; and the capacity to give and receive care (Wesselman, 2012).

##### Assessing differentiation

To establish functional relationships and enjoy psychological wellbeing, it is essential to learn to differentiate ourselves from others. In the context of this article, differentiation refers to an active process in which the patient can connect to their thoughts, feelings, and values while also being close to someone. If the dynamics in the family of origin were healthy, differentiation will have likely taken its natural course and it will not be necessary to intervene. In fact, this differentiation will likely serve as an important resource for the patient. On the other hand, if the process of differentiation did not develop appropriately, it will be common to find confusing dynamics in the family history where everything is blended as well as current difficulties in interpersonal relationships. Part of the therapeutic process will consist of helping the patient gain awareness of these difficulties, while also helping them differentiate from their family of origin. The latter will involve the dissolving of triangulations in the family system that are maladaptive (Kaslow, 2012).

##### Emotional environment and early experiences

Using the *EARLY Scale* (Gonzalez-Vazquez et al., 2019) may be useful to gain information about the possible traumatic experiences and deficiencies that may have been present during the patient’s upbringing. Together with the patient, the therapist would explore how these could have had a negative impact in the patient’s emotional development, interpersonal interactions, and sense of self.

Therapists could then explore the patient’s early experiences with peers. These types of experiences as well as how the family of origin responded to them can significantly shape the beliefs the patient develops about oneself, others, and general human relationships. Therefore, it will be important to identify if this type of memory is present, stored dysfunctionally, and whether they are at the root of the patient’s current presenting concerns.

##### Dysfunctional schemas in intimate partner relationships

Schemas are the emotional and cognitive patterns generally learned early in life, which continue to repeat themselves throughout life. Young (1990, 1999) *Schema Therapy* was designed for individuals who suffer from chronic conditions. Schemas are also likely to be present in non-clinical populations (Young et al., 2013). The concept of schemas can help us to identify the aspects of the patient’s personality that require intervention,organize the information we obtain and present it to the patient without their feeling pathologized or judged. From an EMDR perspective, identifying the patient’s schemas facilitates our ability to classify memories by themes that are at the base of their difficulties and that tend to repeat themselves. This may bring clarity to both patient and therapist.

##### Lacking experiences

Unconsciously, some individuals try to satisfy unmet infant needs in the present. The cognitive and emotional schemas that generate difficulties in adult relationships emerge from these unmet needs (Young et al., 2013). From the point of view of *Schema Therapy*, the following core emotional needs are noted: (a) bonds with others (including safety, stability, care, and acceptance); (b) self-esteem, competence, and sense of self; (c) freedom to express valid needs and emotions; (d) spontaneity and playfulness; and (e) realistic boundaries and self-control. Apart from these needs, it is important to explore if the patient felt invisible in the relationship with the primary caregivers as well as the feelings associated with the effects of that invisibility (Mosquera, 2018).

In relationships where there has been violence or abuse, it is possible that the perpetrator of the violence has used what was lacking in childhood to hook the victim through apparent satisfaction. If there is ambivalence to leave the damaging relationship, it could be useful to explore what the patient considers that their partner has contributed and which no other or very few had contributed before.

#### Prior partner relationships

It is beneficial to identify patterns that are repeated in the different relationships and that become problematic for the patient, their partners, and/or the relationship itself.

##### Role of power in relationships

In this BUAP approach we encourage the therapist to observe if certain patterns around issues of power repeat themselves in the patient’s relationships with partners. It is possible that the patient needs to subjugate their partner, and to the contrary, ends up subjugating themselves to them. It is also possible that the patient involves themselves in conflictive relationships, in which there is escalation between both members to achieve power. This can lead them to be pray to more extreme situations each time.

##### Dysfunctional behaviors

This entails the identification of what type of behaviors are used by the patient in their relationships to feel better in the short-term but that in the medium and/or long-term generate problems for them. There are three important elements that need to be clarified to better understand the behaviors that affect the relationship dynamic: (a) the function that certain behaviors have in the relationship (e.g., controlling the other; protecting them; managing those emotions that activate their attachment bond or difficult subjective experiences; maintaining the relationship or the emotional supply it provides; etc.); (b) the degree of intentionality behind the behavior; and (c) how the patient feels when they rely on these behaviors (e.g., level of awareness in utilizing a dysfunctional behavior and whether there is fault in it or not.)

##### Unrealistic expectations about couples’ relationships

The beliefs that affect couples’ relationships are influenced by what is learned in the social and family context and by what the patient experienced throughout their development. Concretely, within the family environment, the patient may have acquired beliefs through internalization of certain comments or through direct observation of interactions between caregivers. Amongst these beliefs we can encounter myths about romantic love (i.e., ideal relationship with a partner to which we must all aspire), or myths that represent an idealized belief about finding the “adequate partner” or “other-half” who will make everything simpler including the relationship as well as life itself. Another myth may include a patient’s belief of being incomplete until they find a person they can partner with; they see having a partner as a requisite to feeling fully satisfied. An added difficulty about these myths is that they make way for abusers to manipulate their partners. In turn, the partners have internalized that romantic love takes place as a series of behaviors that are clearly dysfunctional for both members and are disempowering of the victim.

##### Need for control

This is one of the facets of personality that can give way to the most damaging aspects of a relationship. Shapiro (2012) orients us to the origin and treatment of this difficulty: “A variety of dysfunctional relational behaviors can be based on childhood experiences that in some way initially gave the individual a sense of security or control… The AIP model encourages therapists to process memories of the interactions that established these patterns (p. 32).” Wesselman (2012) explains that in some cases, control may be the only way a vulnerable person finds to relate to others. This way of behaving, learned early in life, is habitually maintained in adult relationships; especially in those with intimate partners.

#### Negative memories of the distant past and triggers

Through identification of unprocessed memories and their present triggers, the therapist will find some of the targets which may be used when intervening using the standard EMDR protocol.

##### Negative memories

Negative memories having the greatest impact on the patient’s life need to be identified; particularly in reference to their presenting concerns. This may be done either through the symptomatology these memories create when they are dysfunctionally stored; or by the functional limitations they generate day-to-day (i.e., what they activate with the associated negative beliefs.)

##### Triggers

These are identified by exploring instances that generate problems in the patient’s relationships and that can cause them discomfort, block them, or emotionally activate them in a way that reduce their capacity to function in adaptive ways.

#### Resources and limitations in meeting future partners and maintaining relationships

One of the goals of the intervention, will be for the patient (if they agree) to have healthy relationships in the future. Once the patient overcomes the grief process (at least the most complex phases of it), it will be time to inquire about the things that went wrong in previous relationships; what they want and do not want in future partnerships; why they are unable to achieve this; and what happens in the relationships they dislike or that generate problems for them. For some people, meeting a new partner can be quite challenging for various reasons. Explore the difficulties or concrete deficiencies that the patient presents with by exploring the following characteristics.

##### Social skills

Patients whose relationship difficulties are due to deficits in social skills, may be helped to develop them through specific psychoeducation. This will allow the patient to practice these skills in real life while we can process the interactions in therapy. On occasion, the interactions occurring during therapy itself become an ideal opportunity to empathically point out some of the behaviors that are likely to generate difficulties in social situations.

##### Fears and/or avoidance

If the expressed difficulty is that the patient feels anxious, particularly in social situations, the therapist might want to offer psychoeducation about anxiety and fears. It is likely that there will be a need to identify, desensitize, and process the experiences that mediate the anxiety and those that have worsened it. In some patients, the predisposition to anxiety could be due to biological alterations, which also need to be explored (Bulbena, 2016). The latter may require us to refer the patient to a prescribing professional.

At the right moment, use the future template to prepare the patient for exposure and to face real life. Once they have been exposed to their feared social situations, it will become possible to process the triggers that are activated in their interactions with others using BLS. Moreover, efforts need to be made to understand and address the origin of difficulties that start to emerge.

##### Patterns of interaction with others

To understand the patterns of interaction with others, we can rely on *Schema Therapy*, since these patterns are likely related to early development schemas. EMDR is used to identify beliefs, unprocessed emotions, and memories associated to these schemas; hence, to the patient’s patterns of interaction.

##### Communication style

Good communication is fundamental in any human relationship, particularly in intimate relationships. We must identify the patient’s communication style while helping them gain awareness of the dysfunctional strategies that they use (e.g., indirect communication and the impact it has in relationships.) Offer positive alternatives of communicating their needs and address conflict.

##### Empathy and mindsight

Many difficulties in intimate relationships are related with a disparity in the level of empathy between both partners. Empathy has a significant influence in how we perceive others as well as how we interact and relate to them. With individuals who have low empathy, we can intervene to improve their mindsight – defined as one’s capacity to see one’s own mind in a compassionate and conscious way while also imagining the minds of others with care and empathy (Siegel, 2012). The exception to this type of intervention would be, individuals with marked psychopathic features who have traditionally been considered untreatable (Cleckley, 1976). On the other hand, when there is an excess of empathy, it will be important to help the patient differentiate themselves from others. Such patients would benefit from being educated in ways to protect themselves by establishing adequate boundaries while noticing and satisfying their own needs. This may imply working to help the patient overcome barriers to attaining that satisfaction (e.g., not feeling deserving, or feeling guilty, when they put their benefit or well-being before that of others.)

## EMDR phase II: Preparation

In this phase, the therapist offers the patient the necessary resources to adaptively engage in the therapeutic process by providing them with adequate psychoeducation so they can understand their experiences, and if necessary, re-conceptualize their difficulties. This phase, and the following phases, do not necessarily need to be sequential. This is because, frequently, the processing of memories will be accompanied by concurrent psychoeducation.

During this phase, there are two common themes the therapist addresses in a couple’s break-up: (a) any ambivalence about the relationship break-up; and (b) grief related to the loss of the bond and related emotions. This process might be somewhat complicated due to the patient’s personal history; the relationship journey and break-up; limited personal resources; and the patient’s current circumstances. While it is important to not pathologize grieving that follows its natural course, we can always offer support and psychoeducation to normalize their experience and validate emotions associated to it. When grief is complex, it may be necessary to intervene using a more individualized case conceptualization and interventions.

### Installation of resources

To begin working through this phase of EMDR, it is necessary to offer helpful tools to manage any disturbance that may affect the patient during or after therapy sessions (Shapiro, 2012). To do so, we first install one of the resources that we most frequently use in EMDR: the safe place (Shapiro, 2001; Korn and Leeds, 2002). Adaptive information may be introduced to complement this resource, such as psychoeducation on how this supports emotional regulation. If the patient has difficulties working on this type of exercise or the tool does not bring them a sense of calm, alternatives would need to be used.

Often, it is sufficient to install this resource to be able to begin processing stored dysfunctional memories using the procedure that is most adequate for each patient. It is most common that what is most stabilizing for the patient is to be able to process those memories related with emotional hyperarousal and intrusions. If you notice that there is a deficit in emotion regulation, prior to or concurrently, offer the necessary knowledge and tools to improve those abilities. This is an aspect that you must remember to assess in each patient.

Various options are available for cases where we consider appropriate to install additional resources before processing dysfunctional memories. For example:

If the patient is psychologically weak and/or unable to use their own resources and abilities to self-manage through their external or internal experiences, identify resources that can be installed to make them more accessible. One could include prior situations that they resolved successfully, where they felt in control, or which they were able to handle positively (e.g., previous break-ups from other partners that they have already overcome or any other situation where they felt capable.)
If the patient presents with deficits in concrete areas (e.g., inability to be alone, to regulate their emotions, or of feeling self-control, etc.), identify and install resources that may help them manage those specific difficulties.
If there were temporary difficulties in benefiting from social supports – a fundamental resource – the patient would benefit from the installations of moments where they enjoyed the company of others; where they have felt connected; and/or when others served as adequate support.
If there was difficulty keeping distance from an ex-partner, particularly if there is an addictive component in the maintenance of the relationship, it could be useful to install a moment in the future in which the person has been able to overcome the break-up and be okay (Popky, 1994, 2005; Hase, 2010).

## Processing memories

In each stage of this process, we will describe how to apply the different procedures contained in BUAP. We will need to be flexible and adapt to the needs of the patient as we move through the process. On occasion, even if we are in Stage 2 or 3, it may be timely to resort to interventions from Stage 1.

### Stage 1: Ending the relationship and overcoming the break-up EMDR’s R-TEP

The EMDR Recent Traumatic Episode Protocol (EMDR R-TEP) is an integrative protocol that incorporates and extends existing protocols within a new conceptual framework, together with additional measures for containment and safety (Shapiro and Laub, 2014). Therefore, this protocol can be useful when an individual seeks therapy after a relationship break-up that has been traumatic and in which they are just beginning to consolidate memories in their mind. The following are the main advantages in using R-TEP after a break-up from an intimate partner relationship:

It facilitates approaching memories associated with the break-up since they are still being consolidated, so that the different episodes have not been integrated among the rest of the memories (Shapiro, 2013).
It allows the therapist to work with what is emerging in the patient’s mind; what they are prepared to confront; and what is most accessible to them. They do not base the work on their hypotheses about what is the best route for intervention. Instead, therapists allow the patient’s adaptive information system to guide the process under their supervision. Nevertheless, the therapist needs to be attentive to the difficulties and needs that emerge during processing and between sessions.
It gives way to work on the most intrusive material and that which produces the most discomfort or interference in everyday life. This helps the patient in their day-to-day and eases their ability to remain focused and motivated during the therapy sessions.
It addresses what is most distressing to the patient, which tends to result in quick relief. This allows the patient to use resources they could not initially access given the intensity of the emotional and physiological activation as well as the focus on the relationship loss.

When we intervene using R-TEP, the patient narrates the event fully while the therapist only uses BLS during the first processing session. However, when working with a couple’s break-up, it may be useful to repeat this part of the protocol at different times during the therapy process. This will help the patient build a narrative about the relationship and its break-up. The intervention, while therapeutic by itself, also allows us the therapist to identify the changes in that narrative; the aspects of the patient’s experience that they are not able to fully integrate; and those things they learned from the patient’s own history that are maladaptive.

#### Idealization: Processing dysfunctional positive affect

To understand the relevance of working with idealization in EMDR, it is important to develop an understanding of memory processing in a wider sense. Therapists need to not only consider as dysfunctionally stored memories those that encompassed negative events, but also those that included dysfunctional positive affect (Knipe, 2010; Mosquera and Knipe, 2017; Mosquera, 2022).

On occasion, patients will present with idealized mental representations. These may be the image of a moment with positive components; a fragment of a memory; or the memory itself. In the construction of the idealized image, different factors may contribute. These can be individual or sociological, but most important is the defense of *idealization* – a mechanism of psychological protection and survival which is habitually found in individuals who grew up in families whose caregivers were not sufficiently good. This defense is automatically learned during childhood to tolerate the intolerable because escaping or fleeing are not realistic options. This mechanism plays a role in the construction of the idealization of a partner. It will often block access to adaptive information and diminish the patient’s capacity to make healthy decisions. Moreover, the partner can also contribute to activating the creation of an image that is not very realistic of self (Mosquera and Knipe, 2017, 2019; Mosquera, 2022). Below, we list some cases in which it is common to create an idealized image of the partner:

People who are in the falling in love phase of the relationship, in which idealization of the partner tends to predominate.
Those who tend to idealize their partners because they have internalized myths about romantic love, in which they base their relationships.
Individuals who learned to idealize their caregivers as a defense mechanism in their childhood. This, in turn, is reenacted in their adult relationships.
People immersed in relationships with partners with psychopathic or malignant narcissistic profiles. These individuals have deliberately managed to accomplish having their partners (i.e., the patient) internalize an idealized image of them through manipulation and deceit.

When a patient grows up with abusive caregivers, the development of this idealization defense can make the individual more vulnerable to violence and/or abuse from others. And, when these patients partner with someone who has similar dysfunctional patterns to those of their childhood caregivers, the defense of idealization will be unconsciously activated. In turn, this will make it difficult for the patient to identify that they are in an emotional trap that impedes them from taking effective measures to distance and protect themselves.

The idealized image can complicate the grieving process that is associated with a relationship breakup; especially if there has been violence or abuse. The idealization is often more damaging in cases where the victim shows ambivalence to leave the relationship (Mosquera and Knipe, 2017). In victims of violence and/or abuse it may be psychologically exhausting to deal with the internal conflict that the partner’s two opposing images generates: the idealized image and that which is more realistic. Both images are completely incompatible; particularly when the psycho-biological bond that brings them close to the partner who victimizes them is more intense.

To process the idealized image, we will target the “best moment” by using a procedure developed to help women target the ambivalence that can be present when leaving an abusive partner; specifically, this procedure targets the idealization and dysfunctional positive affect (Mosquera and Knipe, 2017; Mosquera, 2022).

#### Subjective sensation of addiction to the relationship

On occasion, what keeps a patient from bringing a relationship to a close or from overcoming a breakup (whether wanted or not), is the urgency to contact the partner. This could be because of pressure to restore the closeness or because of a desire to recover an aspect of the relationship (e.g., physical intimacy).

The sensations and impulses that are described by the individual are like those that we see in an addiction; as such, they can generate feelings of guilt and confusion. When there is a subjective sensation of addiction, it will be beneficial to adopt Shapiro’s perspective that an addiction is stored in the nervous system, and it is not sufficient to make a cognitive decision to overcome it (Shapiro, 2013). Explaining this to the patient can help normalize and understand the difficulties they have in keeping distance from their ex-partner. We can find various cases where the subjective sensation of addiction makes it difficult to break a damaging relationship or accept a break-up that wasn’t chosen. Below, are a few of those instances:

Individuals who find themselves in the falling in love phase of the relationship, since this is one of the most powerful impulses for human beings (Fisher et al., 2006).
Those who are in co-dependent relationships.
People who have been victimized by their partner or ex-partner, who in turn has psychopathic traits. When this is the case, the partner can have a strong hold on the victim through powerful seduction during the initial phase of the relationship. This may include an objectified use of sex and different manipulative tactics (Piñuel, 2016).
Individuals addicted to physical intimacy with their partner.

Patients who present with an intense impulse to reconnect with their ex-partner (even when inconvenient or difficult to do so), will need us to intervene using BLS either on the addictive memory or the implicit memory of positive affect (manifested in their awareness as the impulse to start and repeat the addictive sequence; Hase, 2006.)

To begin processing the addictive memory, we will select one of the components of the addictive behavior as target one. This will be done in function of its accessibility, the patient’s preference, and what they consider safer (Mosquera and Knipe, 2017). Aspects of the addictive memory that may be processed include: desensitizing the urgency to start the addictive behavior (Popky, 2005; Hase, 2006); targeting the positive sensation that accompanies the addictive behavior (Popky, 1995; Knipe, 1998, 2005, 2010; Miller, 2010); desensitizing the anxiety and shame that comes after engaging in the dysfunctional compulsive behavior (Greenwald, 2000); or targeting relapses in addiction (Hase, 2006).

### Stage 2: Working on impediments to maintain healthy relationships

Once the patient can end the relationship and make progress in their grief process, it will be appropriate to conduct a deeper evaluation; particularly, if aside from overcoming the break-up, their goal is to improve future intimate partner relationships or their difficulties with attachment. In doing so, we need to explore why the patient has difficulties in relationships with partners and how they would like to behave in future relationships. Below, is a list of possible foci for the proposed intervention. You may use these for all cases during this stage:

When there are core attachment difficulties, the patient does not just present problems in the last break-up but is also likely to have experienced difficulties in previous relationships. A concrete case is what Nancy Knudsen (2012) coined as chronic relational dysfunction – defined as “the experience of those who are capable of finding and maintaining a healthy intimate partner relationship but who feel considerable emotional angst about the relationship.” The author attributes this to a low level of differentiation (Bowen, 1978), which on occasion will require an intervention on the relationships with the family of origin and the establishment of healthy boundaries.
If the patient cannot care for their own needs or they are not realistic or adaptive, the therapist will help them: identify their needs; evaluate whether they are adaptive or not; and find the way of satisfying them while finding balance between their needs and those of others.
If adequate, proceed with psychoeducation to help the patient achieve capacities and abilities that the patient was not able to develop at the adequate developmental moment due to an insecure attachment.
If difficulties with emotion regulation persist, we can introduce stabilization tools and/or process the memories that are at the source of these difficulties. These types of memories can be negative, but we can also find some that the patient considers to be positive when in fact, they are not. For example, when the patient has grown up in a family where rage explosions and violence are normalized and even valued, a sensation of power and control is manifested in the individual and needs to be resolved.
If the attachment was insecure or damaged, work to repair it by using the EMDR standard protocol on the “attachment injuries” of the individual. Attachment injuries are those that take place when “a significant figure, parent or partner, do not respond during a critical time of need” (Moses, 2012, p. 183).
Work on the traumatic experiences from the last relationship or previous relationships if they have not surfaced during R-TEP or if it’s necessary to deepen exploration of them.
Address any associated memories to the patient’s schemas that may be making it difficult for them to enjoy healthy relationships. We can arrive at these memories through the negative beliefs or the patterns of dysfunctional interactions that they repeat in relationships. For example, looking for partners who are emotionally unavailable.
Finally, work on current triggers.

Additionally, in cases where there has been violence and/or abuse

If it becomes clear during the previous stage that the patient has vulnerabilities that make them susceptible to others being violent or abusive towards them, working on these vulnerabilities will need to be prioritized.
If necessary, provide psychoeducation about where responsibility resides in instances of violence and/or abuse so that the patient can rid themselves of any maladaptive guilt they may feel. This will also help the patient assume the responsibility that belongs to them in the process of recovery. For example, defining what they can do now, and in the future, to manage interpersonal relationships while concentrating on those aspects that depend on them.

### Stage 3: Working with new intimate partner relationships

In this last stage, the goal will be to resolve any difficulties introduced by the patient (if they exist) to meet, initiate, and maintain relationships with new partners. When an individual does not even attempt to establish a relationship with an intimate partner, the main and most common problem is the use of avoidance as a resource to not suffer harm. This behavior may be present because the patient developed a social phobia or due to other motives (e.g., having left a traumatic relationship; not being able to trust others; being fearful of choosing an inadequate partner or someone who mistreats them.)

We may also find individuals who have difficulties managing some of the stages of the relationship. For example, some may become overwhelmed by the emotions associated with falling in love or intimacy; others feel ambivalent; and yet others fear they will repeat hurtful patterns or present with a deficit of abilities. In these cases, the goal will be to accompany the patient in the stage of the relationship that is difficult for them. Easing exposure to those aspects that they fear by offering adequate psychoeducation and using EMDR to process triggers that are activated will be beneficial.

When despite the work on the previous stages the patient continues to experience insecurity and doubts in facing a feared situation, the first step will be to look for related memories that have not been previously identified. Once these have been processed along with present triggers, working on a future template will follow. This can be repeated as many times as necessary so the person can solve in their imagination the feared situation in the most effective manner. After applying the future template, we expect that the person will be able to put into practice, in the real world, what they were avoiding or inadequately facing. The latter may help us identify additional aspects to process (Shapiro, 2012).

Finally, work with the abilities that the patient needs to meet new people and maintain healthy relationships. Shapiro (2012) best explained it: “It is much easier for patients to improve their social skills if they do not have old unprocessed memories that make them feel insecure and defective. Consequently, EMDR works from the inside out. It takes care of the internal world before using the excellent modeling tool – *experiential roleplaying tool* - to incorporate a set of skills that help define a healthy adult (p. 32).”

## Conclusion

The guidelines proposed in this article to intervene with patients who seek therapy after the break-up from a relationship with an intimate partner, have been introduced with the purpose of addressing the concrete difficulties individuals experience in their process of grief and from the loss of their secure base. These relationship break-ups can interfere with many of the patients’ daily experiences and lead them to even abandon therapy if it is not adapted to address the factors that bring them the most discomfort. The proposed framework consists of a series of adaptations of EMDR’s standard protocol while keeping in mind the frequent difficulties in these relationship break-ups.

The stages of intervention introduced here are simply a guide or framework to follow. Variations and adaptations must be individualized. It will not be necessary to follow all the proposed steps in this article. Rather, the intervention should be adapted to the difficulties that show up at different moments of the therapeutic process.

## Author contributions

All authors listed have made a substantial, direct, and intellectual contribution to the work and approved it for publication.

## Conflict of interest

The authors declare that the research was conducted in the absence of any commercial or financial relationships that could be construed as a potential conflict of interest.

## Publisher’s note

All claims expressed in this article are solely those of the authors and do not necessarily represent those of their affiliated organizations, or those of the publisher, the editors and the reviewers. Any product that may be evaluated in this article, or claim that may be made by its manufacturer, is not guaranteed or endorsed by the publisher.
